# A Specific microRNA Targets an Elongase of Very Long Chain Fatty Acids to Regulate Fatty Acid Composition and Mitochondrial Morphology of Skeletal Muscle Cells

**DOI:** 10.3390/ani12172274

**Published:** 2022-09-02

**Authors:** Han Wang, Moran Hu, Zhonghao Shen, Xiaolong Zhou, Songbai Yang, Ke He, Xiangchen Li, Feifei Yan, Ayong Zhao

**Affiliations:** Key Laboratory of Applied Technology on Green-Eco Healthy Animal Husbandry of Zhejiang Province, College of Animal Science and Technology, College of Veterinary Medicine, Department of Animal Science, Zhejiang A&F University, Hangzhou 311300, China

**Keywords:** miR-22, elongation of long-chain fatty acids family member 6 (ELOVL6), fatty acid composition, mitochondrial morphology, C2C12

## Abstract

**Simple Summary:**

Recently, it was found that miR-22 might be involved in the regulation of muscle tenderness and was an important miRNA that affects meat quality. In the present study, we demonstrated that miR-22 regulates the fatty acid composition and mitochondrial morphology of skeletal muscle cells by inhibiting ELOVL6 expression. This study presented the first systematical investigation on the miR-22 correlation with fatty acid composition and mitochondrial function in muscle cells. Our finding is important to help us to understand the function of miR-22 in skeletal muscle cells and the complicacy of muscle metabolism regulation.

**Abstract:**

Recently, miR-22 has been suggested to be an important microRNA (miRNA) affecting meat quality. Studies have shown that muscle fatty acid composition and mitochondrial function are closely related to meat quality. The regulatory mechanism of miR-22 on skeletal muscle fatty acid composition and mitochondrial function is not well characterized. Therefore, we aimed to explore the effects of miR-22 on fatty acid composition and mitochondrial function in C2C12 cells. Here, it demonstrate that elevated expression of miR-22 significantly repressed fatty acid elongation and mitochondrial morphology in C2C12 myoblasts, while the knockdown of miR-22 showed opposite results. Furthermore, miR-22 targets the elongase of very long chain fatty acids 6 (ELOVL6) and represses its expression in muscle cells. Knockdown of ELOVL6 mimicked the effect of miR-22 on fatty acid composition and mitochondrial function, while overexpression of ELOVL6 restored the effects of miR-22. These findings indicate that miR-22 downregulates the elongation of fatty acids and mitochondrial morphology by inhibiting ELOVL6 expression in muscle cells, which may provide some useful information for controlling muscle lipid accumulation and mitochondrial function in livestock in the future.

## 1. Introduction

Muscle fatty acid composition and mitochondrial function are important in pork quality [1,2]. Studies have found that the tenderness, flavor, and juiciness of pork are positively correlated with the contents of monounsaturated and polyunsaturated fatty acids [3]. Oleic acid is one of the major monounsaturated fatty acids (MUFAs) in meat and conveys a favorable tenderness and good quality to the meat [4,5]. In addition, the number and structural integrity of mitochondria in muscle affect oxidative metabolism, which further affects meat tenderness and color [6]. Therefore, exploring the molecular regulation of fatty acid composition and mitochondrial function in muscle cells is of great significance for improving meat quality.

As small non-coding RNAs that are involved in gene regulation, microRNAs (miRNAs) also play a key role in regulating meat quality traits such as muscle development, drip loss, and meat color [7,8]. As previously established, miR-22 was highly expressed in muscle and regulated myoblast proliferation and differentiation by targeting Transforming Growth Factor Beta Receptor 1 (TGFBR1) [9]. miR-22 may also be involved in the regulation of muscle tenderness and has been shown to be an important miRNA affecting meat quality [10]. We initially posited that miR-22 might affect meat quality by regulating the different types of muscle fibers. However, a recent study showed that muscle fiber type composition and mitochondrial content in the muscles of miR-22 knockout mice was unaffected, although the oxidation of peroxisome fatty acids outside the mitochondria, and β-oxidation inside the mitochondria, increased significantly. [11]. Therefore, the effects of miR-22 on meat quality might be related to fatty acid composition and mitochondrial function. However, the regulatory mechanism of miR-22 on skeletal muscle fatty acid composition and mitochondrial function is not well characterized.

Elongation of long-chain fatty acids family member 6 (ELOVL6) is an elongase that catalyzes de novo synthesis of fatty acids [12]. It primarily catalyzes the extension of C12-C16 saturated or monounsaturated fatty acids, and plays an important role in the conversion of palmitic acid (C16:0) to stearic acid (C18:0), as well as in the elongation of stearic acid (C18:0) to oleic acid (C18:1, n-9) [12]. Although mitochondrial fusion and function have been shown to be inhibited in the *Drosophila melanogaster* ELOVL6 knockout [13], few studies have investigated the involvement of ELOVL6 in muscle fatty acid composition and mitochondrial function. Chicken miR-22 has been shown to affect the expression of the ELOVL6 gene, thereby participating in the regulation of chicken liver lipid metabolism [14]. Therefore, we hypothesized that miR-22 could inhibit skeletal muscle cell fatty acid composition and mitochondrial function by targeting ELOVL6.

In the present study, it was demonstrated that miR-22 increased the content of palmitic acid (C16:0) but reduced that of oleic acid (C18:1) and linoleic acid (C18:2), by inhibiting the expression of ELOVL6 in C2C12 muscle cells. miR-22 also reduced the mitochondrial membrane potential of muscle cells and promoted mitochondrial division and the abundance of abnormal cristae. Additionally, miR-22 targeted the expression of ELOVL6 to inhibit fatty acid composition- and mitochondrial function-related genes. This study aimed to reveal a novel function of miR-22 in skeletal muscle fatty acid composition and mitochondrial function. 

## 2. Materials and Methods

### 2.1. Cell Culture

The C2C12 (mouse) and HEK 293T cell lines (human) used in this study were obtained from the American Type Culture Collection (ATCC, Manassas, VA, USA). C2C12 cells were used to explore gene function in livestock muscle cells [15,16]. C2C12 cells were first cultured in growth medium (GM)—Dulbecco’s modified Eagle’s medium (DMEM, Hyclone, Logan, UT, USA)—supplemented with 10% (volume fraction) fetal bovine serum and 1% penicillin-streptomycin (Gibco, New York, NY, USA). Then, the cells were induced to differentiate in a differentiation medium (DM) containing 2% horse serum and penicillin-streptomycin (50 mg/mL) (Gibco). Subsequent assays were conducted in C2C12 cells, which were differentiated for 6 days. All the samples were collected after 24 h of transfection. The 293T cells were cultured in 24-well plates in DMEM with 10% fetal bovine serum (FBS) and penicillin-streptomycin (50 mg/mL). All cells were incubated at 37 °C under 5% CO_2_.

### 2.2. Quantitative Real-Time PCR

The TRIzol reagent was used to extract total RNA [17]. Briefly, TRIzol (Invitrogen, Waltham, MA, USA) was added to the culture dish to lyse the cells. Then, 0.2 mL of chloroform per 1 mL of TRIzol reagent was added, and the samples were incubated at 15–30 °C for 2–3 min. Samples were then centrifuged for 15 min at 12,000× *g* at 4 °C. Following centrifugation, the aqueous phase was transferred to a fresh tube, and 0.5 mL of isopropyl alcohol per 1 mL of the TRIzol reagent used for the initial homogenization was added. Samples were incubated at 15–30 °C for 10 min and centrifuged at 12,000× *g* for 10 min at 2–8 °C. The supernatant was removed, and the RNA pellet was washed once with 75% ethanol. The RNA pellet was then air-dried for 5–10 min. Integrity, reverse transcription, and quantitative real-time polymerase chain reaction (qPCR) were performed as described previously [18]. The experiment was replicated at least three times for each treatment and control. Differential expression was analyzed using the 2^−ΔΔCt^ method [19]. The primer sequences are provided in Table 1.

### 2.3. Plasmid Construction

For the pcDNA 3.1 ELOVL6 expression vector, the entire coding sequence (CDS) of ELOVL6 was cloned into the HindIII and NheI restriction sites of the pcDNA 3.1 plasmid (Invitrogen). The primer sequences were as follows: 5′-GCTAGCATGAACATGTCAGTGTTGAC-3′ and 5′-AAGCTTCTAGTCAGCCTTGGTGGTT-3′. The Psicheck-2 dual-luciferase reporter and ELOVL6 point mutant vectors were generated by Tsingke Biotechnology Co., Ltd. (Hangzhou, China).

### 2.4. RNA Oligonucleotides and Transfection

MiR-22 mimics, the negative control (NC), miR-22 inhibitor, inhibitor NC, small interfering RNA (siRNA), and siRNA NC were designed and synthesized by RiboBio (Guangzhou, China). Lipofectamine 3000 (Invitrogen) was used for transfection according to the manufacturer’s instructions. The transfections of mimic oligonucleotides were at a final concentration of 50 nM, while the inhibitors were 100 nM. The RNA oligo sequences are shown in Supplemental Table S1.

### 2.5. Dual-Luciferase Reporter Assay

In 12-well plates, miR-22 or NC mimics (50 nM) were transfected into HEK293T cells with 1 μg Psicheck-2 ELOVL6 luciferase vector (wild-type or mutant) using Lipofectamine 3000 (Invitrogen). The assays were performed 24 h after transfection according to the manufacturer’s instructions (Promega, Madison, WI, USA). All experiments were performed in triplicate wells and repeated three times.

### 2.6. Western Blot

Total protein was harvested using radioimmunoprecipitation (RIPA) lysis buffer (Beyotime, Jiangsu, China) with 1% phenylmethylsulfonyl fluoride (PMSF) (Beyotime). Using 12% SDS-PAGE, proteins were separated and transferred onto polyvinylidene fluoride membranes (Millipore, Burlington, MA, USA), which were incubated with primary antibodies against ELOVL6 (ab69857, Abcam, Cambridge, UK) and β-tubulin (Am1031a, Abcepta, Suzhou, China) after blocking with 5% bovine serum albumin. Horseradish peroxidase-labeled anti-rabbit/mouse IgG (Abbkine, Wuhan, China) was used as a secondary antibody. Western blotting results were visualized with the ECL reagent (Thermo Scientific, Waltham, MA, USA) with a chemiluminescence detection system (Tanon, China). The gray value was analyzed using ImageJ software (NIH, Bethesda, MD, USA). The experiment was replicated at least three times for each treatment and control.

### 2.7. Fatty Acid Methyl Ester and Gas Chromatography (GC) Analysis

After 24 h of transfection, cells were collected, washed, and resuspended in methanol. Potassium hydroxide was added, and the samples were incubated in a 55 °C water bath for 1.5 h, with vigorous shaking every 20 min. After cooling below room temperature, sulfuric acid was added, and the samples were incubated at 55 °C for 1.5 h, with shaking every 20 min. N-hexane (1 mL) was added when the sample reached room temperature. The samples were then rotated for 5 min and centrifuged at 4 °C at 1600× *g* for 5 min. The n-hexane layer was finally transferred to a 2 mL centrifuge tube and stored at −80 °C.

An HP-88 column (100 m × 250 μm × 0.2 μm, pn112-88A7, Agilent, Santa Clara, CA, USA) was used with nitrogen as the carrier gas. An ultra-inert split liner (pn5190-2295) was used as the injection port liner. The injection port temperature and the injection volume were 270 °C and 1 μL, respectively. The split injection was adopted with a split ratio of 25:1. The detector temperature was 280 °C and the makeup flow (N_2_) was 25 mL/min, while the column flow rate (N_2_) was 0.9 mL/min and 32 psi constant pressure mode was used. The initial temperature of the heating program was 100 °C for 5 min, which was increased to 170 °C at a rate of 10 °C/min. This temperature was maintained for 8 min, then increased to 200 °C at a rate of 1 °C/min, whereas 200 °C was maintained for 20 min and increased to 230 °C at a rate of 8 °C/min. The final temperature of 230 °C was maintained for 10 min.

A total of 37 fatty acid methyl ester mixed standard products (CRM 47885, Sigma, St. Louis, MO, USA) were weighed and diluted in a standard solution series of 1, 5, and 10 mg/mL by adding chromatographic-grade methanol. The solutions were placed in a sample bottle and assessed based on two parallel determinations, and the peak area was quantified to draw a standard curve. The area normalization method was used to analyze the fatty acid composition (%) [20]. The experiment was replicated at least three times for each treatment and control.

### 2.8. Mitochondrial Membrane Potential Detection

After 24 h of transfection, the culture medium was discarded, and the cells were washed with phosphate-buffered saline (PBS). Then, the cells were trypsinized, and an equal volume of fresh medium was added to stop the digestion. The cells were centrifuged and washed three times with PBS. The test was performed according to the instructions of the mitochondrial membrane potential assay kit (JC-1, Solarbio, Beijing, China). The Synergy-4 multifunctional microplate reader (BioTek, Winooski, VT, USA) was used to detect the fluorescence values at excitation and emission wavelengths of 490 and 530 nm (green), respectively. Subsequently, the fluorescence values at excitation and emission wavelengths of 525 and 590 nm (red), respectively, were detected. Changes in mitochondrial membrane potential were determined by calculating the ratio of red/green fluorescence. The experiment was replicated at least three times for each treatment and control.

### 2.9. Electron Microscopy Analysis

After 24 h of transfection, the culture medium was discarded, and cells were washed with PBS. Then, cells were trypsinized, and an equal volume of fresh medium was added to stop the digestion. The cells were centrifuged, washed three times with PBS, and fixed in 2.5% glutaraldehyde at 4 °C overnight. After rinsing with PBS, the cells were fixed in 1% osmium tetroxide for 1–2 h. Afterward, cells were dehydrated with an ethanol gradient (30%, 50%, 70%, 80%, 90%, and 95%), and cells were treated with each concentration for 15 min. Thereafter, the cells were treated with 100% ethanol for 20 min followed by pure acetone for 20 min. A mixture of Spurr resin (EMS, Ft. Washington, PA, USA) and acetone (1:1 volume ratio) was added to the sample for 1 h. Then, the cells were treated with a 3:1 mixture of Spurr resin and acetone (volume ratio) for 3 h. Samples were embedded in fresh Spurr resin and cut into 70–90-nm sections, which were stained with uranyl acetate and lead citrate. Images were acquired using a JEM-1230 transmission electron microscope (JEOL, Tokyo, Japan). The analysis statistics are based on the scan of the entire slice. A randomly selected point on the slice was magnified and analyzed. Each treatment concluded with three repeats, with at least fifteen points in each repeat.

### 2.10. Statistical Analysis

All data are shown as the mean ± SEM, and unpaired Student’s *t*-tests were used to calculate *p*-values. The analyses were performed with SPSS software (ver. 20.0, SPSS Inc., Chicago, IL, USA). *p* < 0.05 was considered significant and *p* < 0.01 was highly significant.

## 3. Results

### 3.1. miR-22 Represses Fatty Acid Elongation in C2C12 Cells

C2C12 cell lines usually serve as an easy-to-handle model system for investigating the molecular basis of skeletal muscle cell specification and development both in humans and farm animals [16,21,22]

**Figure 1:**
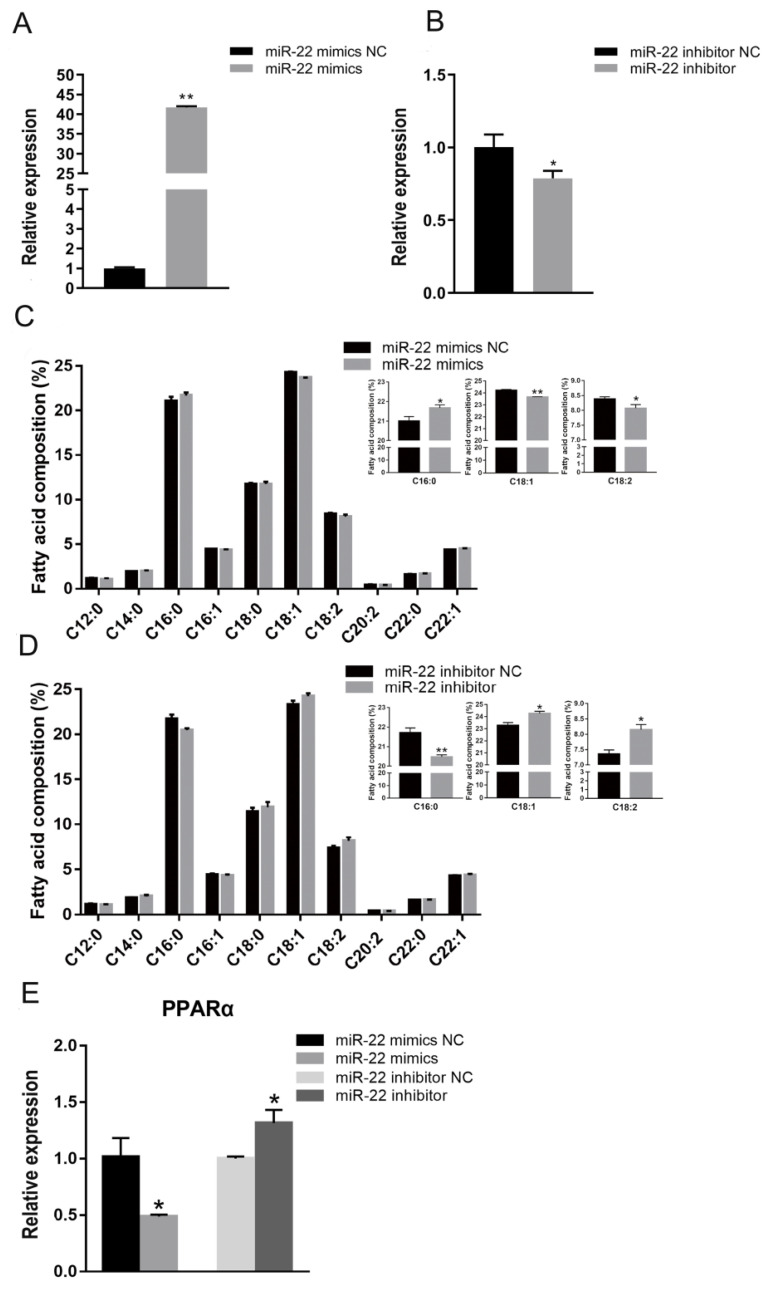
MiR-22 inhibits fatty acid elongation in C2C12 cells. (**A**) The expression of miR-22 was detected using qPCR in myotubes transfected with miR-22 mimics or negative control (NC). (**B**) miR-22 expression was detected in myotubes following transfection with miR-22 inhibitor or inhibitor NC. (**C**) Fatty acid composition was measured after C2C12 cells had been transfected with miR-22 mimics or NC for 24 h. The contents of C16:0, C18:1, and C18:2 were determined. (**D**) Fatty acid composition was measured after C2C12 cells had been transfected with miR-22 inhibitor or inhibitor NC for 24 h. The contents of C16:0, C18:1, and C18:2 were determined. (**E**) Expression of PPARα at 24 h after transfection. * *p* < 0.05, ** *p* < 0.01. Data represent mean ± SEM from at least three independent experiments.

To explore the effects of miR-22 on fatty acid composition in C2C12 cells, synthetic miR-22 mimics or NC were transfected into myotubes, which were cultured in DM for 6 days (Figure 1A,B). GC indicated that miR-22-transfected cells had a higher proportion of palmitic acid (C16:0) than control cells, while the oleic acid (C18:1) and linoleic acid (C18:2) contents were significantly reduced (Figure 1C). The opposite results were observed following the inhibition of miR-22 expression (Figure 1D). Elevated miR-22 expression significantly decreased the mRNA level of peroxisome proliferator-activated receptor alpha (PPARα), a fatty acid metabolism-related gene (Figure 1E). However, inhibition of miR-22 significantly increased the expression of PPARα (Figure 1E). These results indicate that miR-22 can inhibit fatty acid elongation in C2C12 cells.

### 3.2. miR-22 Reduces Membrane Potential but Promotes Division and Abnormal Cristae of C2C12 Cell Mitochondria

**Figure 2:**
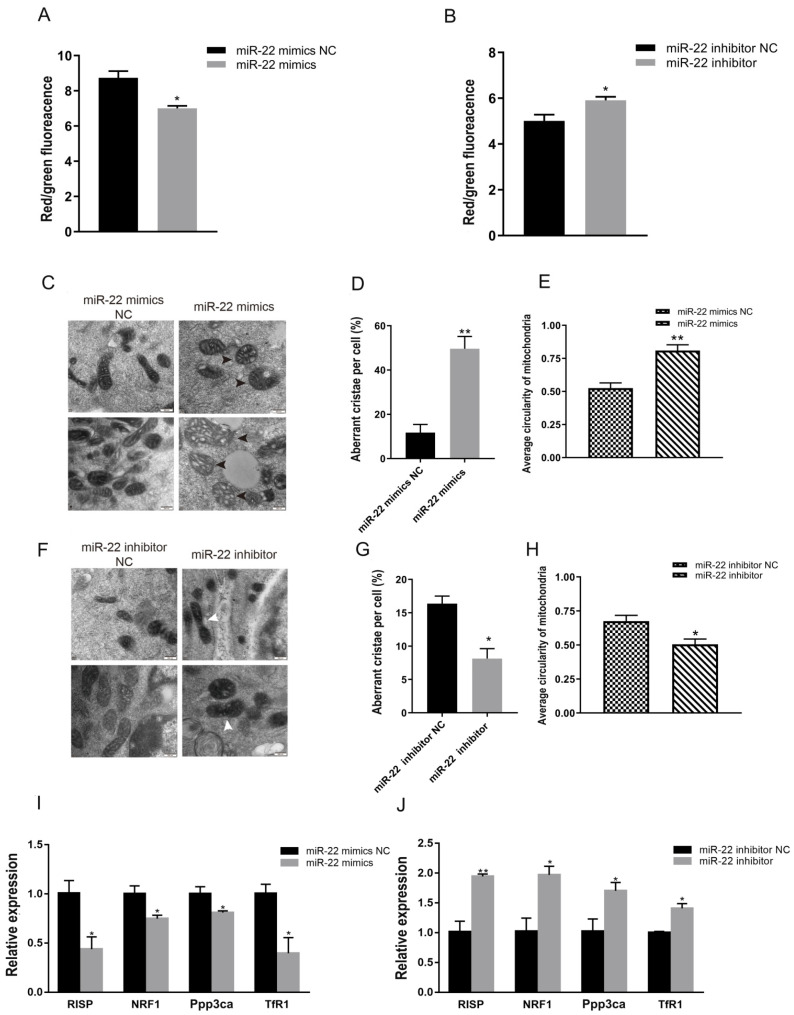
MiR-22 influences mitochondrial morphology in C2C12 cells. (**A**) Mitochondrial membrane potential in C2C12 cells was detected by JC-1 following transfection with miR-22 mimics or negative control (NC). (**B**) Mitochondrial membrane potential in C2C12 cells was detected by JC-1 following transfection with miR-22 inhibitor or inhibitor NC. (**C**) Mitochondrial morphology was observed via transmission electron microscopy (TEM) following transfection with miR-22 mimics or NC. The black arrow shows an abnormal crista. (**D**) The percentage of mitochondria with abnormal cristae in each cell was calculated. (**E**) The average circularity of mitochondria was calculated. (**F**) Mitochondrial morphology was observed via TEM following transfection with miR-22 mimics or NC. The white arrow shows normal fusion of mitochondria. (**G**) The percentage of mitochondria with vacuoles and abnormal cristae in each cell was calculated. (**H**) The average circularity of mitochondria was calculated. Scale bar, 200 nm. (**I**) The expression of mitochondrial function-related genes in C2C12 cells following transfection with miR-22 mimics or NC. (**J**) The expression of mitochondrial function-related genes in C2C12 cells following inhibition of miR-22. * *p* < 0.05, ** *p* < 0.01. Data represent mean ± SEM from at least three independent experiments.

To assess the effect of miR-22 on the mitochondrial morphology of C2C12 cells, the JC-1 method was used to detect changes in mitochondrial membrane potential after transfection. Compared to the NC, overexpression of miR-22 effectively reduced the mitochondrial membrane potential of C2C12 cells (Figure 2A), while knockdown of miR-22 resulted in a dramatic increase in mitochondrial membrane potential (Figure 2B). 

Changes in mitochondrial morphology were also observed. Significant vacuole-like damage occurred in mitochondria following the overexpression of miR-22 (Figure 2C), and the proportion of mitochondria with abnormal cristae increased significantly (Figure 2D). The average circularity of mitochondria was also obviously increased (Figure 2E). However, the proportion of abnormal mitochondrial cristae and average circularity was significantly reduced when miR-22 was inhibited (Figure 2F–H). The expression of marker genes related to mitochondrial function (Rieske iron-sulfur protein (RISP), protein phosphatase 3, catalytic subunit, alpha isoform (Ppp3ca), nuclear respiratory factor 1 (NRFl), and transferrin receptor (TfR1)), was significantly downregulated when miR-22 was overexpressed (Figure 2I). By contrast, miR-22 loss-of-function upregulated the mRNA expression of RISP, Ppp3ca, NRFl, and TfR1 (Figure 2J). Collectively, these data indicate that miR-22 can repress mitochondrial morphology and function.

### 3.3. miR-22 Directly Targets the Elongase of Very Long Chain Fatty Acids 6 (ELOVL6) Gene in C2C12 Cells

**Figure 3:**
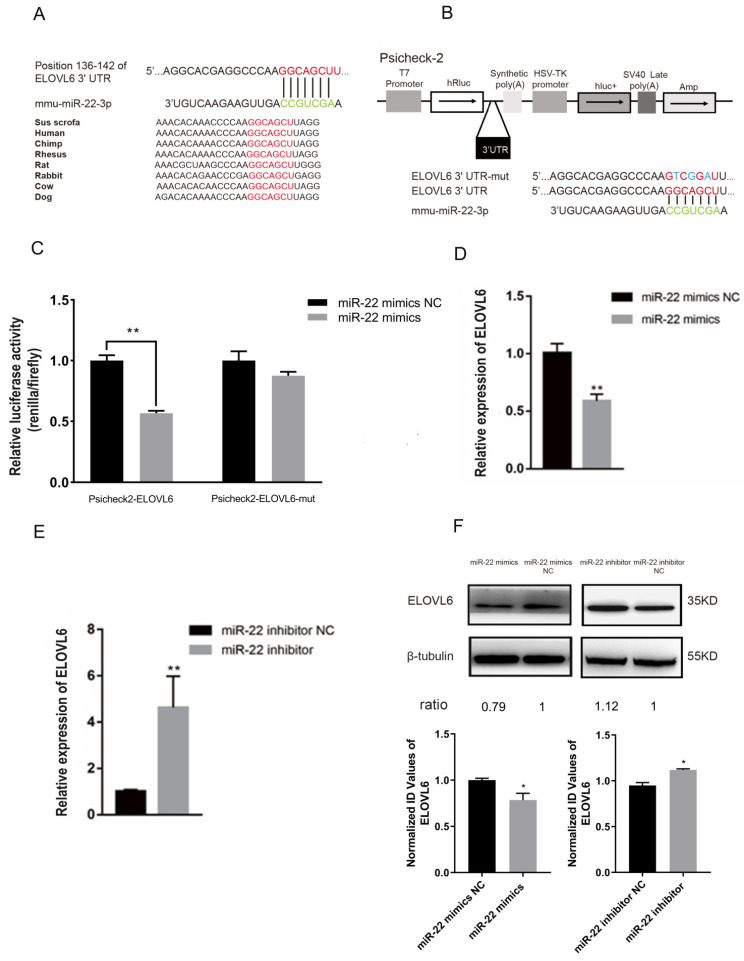
Elongation of long-chain fatty acids family member 6 (ELOVL6) is a target gene of miRNA-22. (**A**) The sequence of miR-22 and its predicted conserved binding region in ELOVL6 3′UTR (red). (**B**) Structure diagram of dual-luciferase reporter vector Psicheck-2. The predicted miR-22 target site of the ELOVL6 3′UTR and mutation target site were inserted into the 3′ end of the Renilla luciferase gene (hRluc). The expression of firefly luciferase gene (hluc+) was used as the standard control. (**C**) C2C12 and HEK293T cells transfected with miR-22 mimics or NC were co-transfected with the Psicheck-2 ELOVL6 or Psicheck-2 ELOVL6-mut vector. Relative luciferase activity was determined after 24 h. (**D**) The expression of ELOVL6 mRNA in C2C12 cells following transfection of miR-22 mimics or negative control (NC). (**E**) The expression of ELOVL6 mRNA in C2C12 cells following transfection of miR-22 inhibitor or inhibitor NC. (**F**) The protein expression of ELOVL6 following transfection with miR-22 mimics, NC, miR-22 inhibitor, or inhibitor NC for 24 h was detected by Western blotting. Ratios represent the densitometry value of the treatment group compared to the control group. * *p* < 0.05, ** *p* < 0.01. Data represent mean ± SEM from at least three independent experiments.

To further investigate the role of miR-22 in fatty acid composition and mitochondrial function in muscle cells, the miR-22 target genes were predicted by the online prediction program TargetScan. ELOVL6 was a predicted target of miR-22 and has a miR-22 binding site in the 3′UTR region, which was highly conserved in multiple species (Figure 3A). Next, a dual luciferase reporter assay was conducted to confirm ELOVL6 as a target gene of miR-22. Using the Psicheck-2 vector, the predicted sequence of the ELOVL6 3′UTR was inserted into the Renilla luciferase (hRluc) gene 3′UTR sequence (Figure 3B). In addition, the firefly luciferase gene (hluc^+^) was used as a control gene to normalize fluorescence expression (Figure 3B). A Psicheck-2 vector with three mutant sites in the binding site was also generated (Figure 3B). miR-22 mimics or NC were co-transfected with Psicheck-2 ELOVL6 or Psicheck-2 ELOVL6-mut vector into HEK293T cells. Luciferase activity was significantly inhibited, while no significant changes in luciferase activity were observed following the overexpression of miR-22 (Figure 3C). These results indicate that ELOVL6 is a target of miR-22.

The role of ELOVL6 in the regulation of miR-22 was also considered in muscle cells. Following the overexpression of miR-22 in differentiated C2C12 cells, ELOVL6 gene expression was downregulated (Figure 3D,F). However, ELOVL6 expression was significantly upregulated after tthe knockdown of miR-22 (Figure 3E,F). Thus, miR-22 plays an important role in suppressing ELOVL6 expression in C2C12 cells.

### 3.4. ELOVL6 Has a Positive Effect on Fatty Acid Elongation

**Figure 4:**
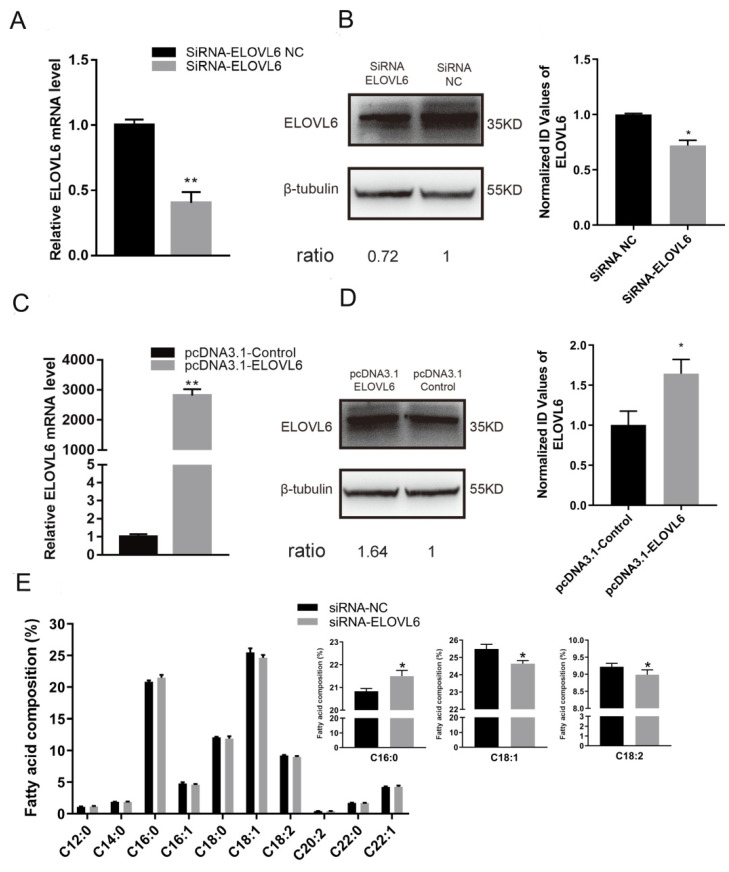

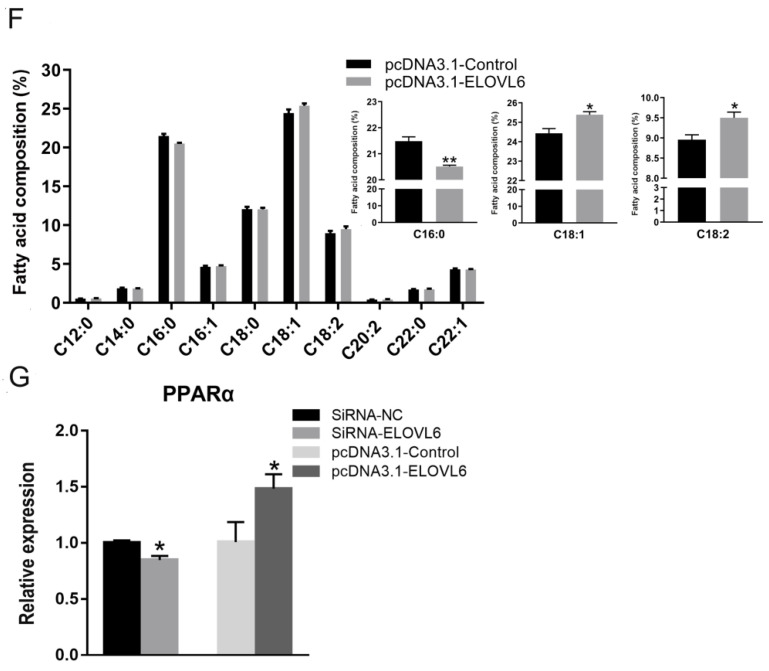
Elongase of very long chain fatty acids 6 (ELOVL6) promotes fatty acid elongation in C2C12 cells. (**A**) Detection of ELOVL6 mRNA expression in C2C12 cells by qPCR following transfection with siRNA-ELOVL6 or negative control (NC) for 24 h. (**B**) Detection of ELOVL6 protein expression in C2C12 cells by Western blotting following transfection with siRNA-ELOVL6 or NC. Ratios represent the densitometry value of the treatment group compared to that of the control group. (**C**) Detection of ELOVL6 mRNA expression in C2C12 cells by qPCR following transfection with pcDNA3.1 ELOVL6 or pcDNA3.1 control. (**D**) Detection of ELOVL6 protein expression in C2C12 cells by Western blotting following transfection with pcDNA3.1 ELOVL6 or pcDNA3.1 control. Ratios represent the densitometry value of the treatment group compared to the control group. (**E**) Fatty acid composition was measured after C2C12 cells had been transfected with siRNA-ELOVL6 or NC for 24 h. The contents of C16:0, C18:1, and C18:2 were determined. (**F**) Fatty acid composition was measured after C2C12 cells had been transfected with pcDNA3.1 ELOVL6 or pcDNA3.1 control for 24 h. The contents of C16:0, C18:1, and C18:2 were determined. (**G**) The expression of perixisome proliferation-activated receptor alpha (PPARα) at 24 h after transfection with siRNA-ELOVL6, NC, pcDNA3.1 ELOVL6, or pcDNA3.1 control. * *p* < 0.05, ** *p* < 0.01. Data represent mean ± SEM from at least three independent experiments.

The anti-ELOVL6 siRNA and the pcDNA 3.1 vector were used to knockdown and upregulate the expression of ELOVL6, respectively, to elucidate its role in muscle cell fatty acid composition (Figure 4A–D). The siRNA group had a higher proportion of palmitic acid (C16:0) than the NC group (Figure 4E), while oleic acid (C18:1) and linoleic acid (C18:2) contents were decreased (Figure 4E). The overexpression of ELOVL6 showed opposite results (Figure 4F). The mRNA level of PPARα was significantly downregulated when ELOVL6 was inhibited (Figure 4G). However, an apparent increase in PPARα expression was observed when the expression of ELOVL6 was upregulated (Figure 4G). These outcomes suggest that ELOVL6 accelerates fatty acid elongation in C2C12 cells. 

### 3.5. ELOVL6 Promotes Cell Membrane Potential and Maintains Normal Mitochondrial Morphology of C2C12 Cells

**Figure 5:**
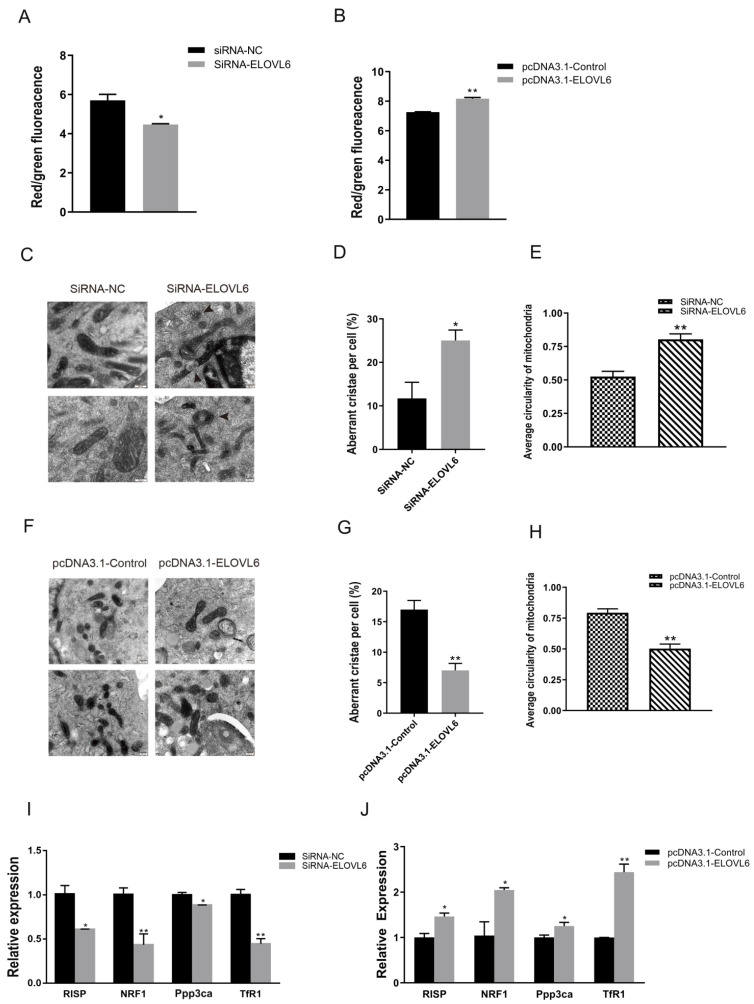
ELOVL6 affects the mitochondrial morphology in C2C12 cells. (**A**) The mitochondrial membrane potential in C2C12 cells was detected by JC-1 following transfection with siRNA-ELOVL6 or negative control (NC). (**B**) The mitochondrial membrane potential in C2C12 cells was detected by JC-1 following transfection with pcDNA3.1 ELOVL6 or pcDNA3.1 control. (**C**) Mitochondrial morphology was observed by transmission electron microscopy (TEM) following transfection with siRNA-ELOVL6 or NC. The black arrow shows an abnormal crista of the mitochondria. (**D**) The percentage of mitochondria with abnormal cristae in each cell was calculated. (**E**) The average circularity of mitochondria was calculated. Scale bar, 200 nm. (**F**) Mitochondrial morphology was observed by TEM following transfection with pcDNA3.1 ELOVL6 or pcDNA3.1 control. (**G**) The percentage of mitochondria with vacuoles and abnormal cristae in each cell was calculated. (**H**) The average circularity of mitochondria was calculated. (**I**) The expression of mitochondrial function-related genes in C2C12 cells following transfection with siRNA-ELOVL6 or NC. (**J**) The expression of mitochondrial function-related genes in C2C12 cells following transfection with pcDNA3.1 ELOVL6 or pcDNA3.1 control. * *p* < 0.05, ** *p* < 0.01. Data represent mean ± SEM from at least three independent experiments.

To explore the effect of ELOVL6 on the mitochondrial morphology of C2C12 cells, the ELOVL6 siRNA and the pcDNA3.1 vector were transfected into differentiated C2C12 cells. The mitochondrial membrane potential of C2C12 cells decreased significantly in the siRNA-treated group (Figure 5A). However, a significant increase in mitochondrial membrane potential was observed when ELOVL6 was overexpressed (Figure 5B). Knockdown of ELOVL6 also increased vacuole-like damage and abnormal cristae in the mitochondria (Figure 5C,D), and the average circularity of mitochondria was also remarkably increased (Figure 5E). Conversely, when the expression of ELOVL6 was increased, the proportion of abnormal mitochondrial cristae and average circularity of cell mitochondria decreased (Figure 5F–H). When the expression of ELOVL6 was repressed, the mRNA expression of genes related to mitochondria function decreased dramatically (Figure 5I), while mRNA levels of these genes were reduced when ELOVL6 was overexpressed (Figure 5J). Thus, these results suggest that ELOVL6 helps maintain mitochondrial morphology and function.

### 3.6. ELOVL6 Compensates for the Effects of miR-22 on Fatty Acid Composition and Mitochondrial Morphology in C2C12 Cells

**Figure 6:**
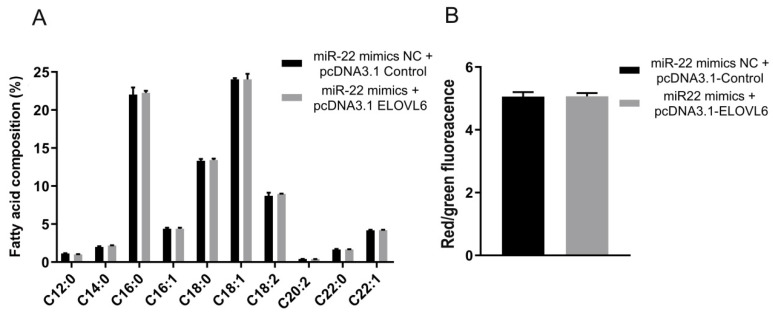

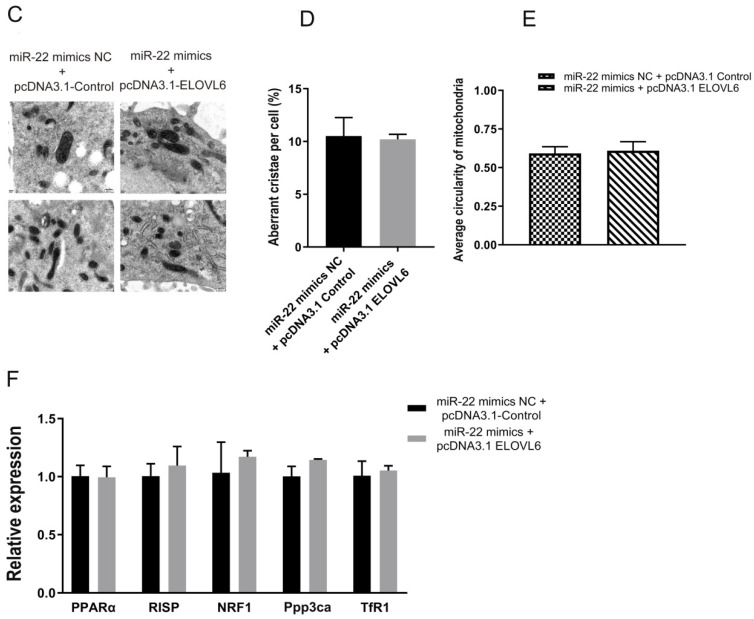
Elongase of very long chain fatty acids 6 (ELOVL6) gene expression reverses the functional changes caused by miR-22. (**A**) Determination of C2C12 cell fatty acid composition by gas chromatography. (**B**) Detection of mitochondrial membrane potential changes by JC-1. (**C**) Mitochondrial morphology was observed by transmission electron microscope (TEM). (**D**) The percentage of mitochondria with abnormal cristae in each cell was calculated. (**E**) The average circularity of mitochondria was calculated. Scale bar, 200 nm. (**F**) The expression of mitochondrial function-related genes in C2C12 cells after transfection. Data represent mean ± SEM from at least three independent experiments.

To further verify that miR-22 can target the ELOVL6 gene to regulate the fatty acid composition and mitochondrial morphology in C2C12 cells, miR-22 mimics and the ELOVL6 pcDNA3.1 vector were co-transfected into differentiated C2C12 cells. The relative contents of palmitic acid (C16:0), oleic acid (C18:1), and linoleic acid (C18:2) in the co-transfection group were not significantly different from those in the control group (Figure 6A). In addition, the mitochondrial membrane potential of cells in the co-transfection group was not significantly different compared to the control group (Figure 6B), and the mitochondrial morphology in the co-transfection group returned to normal (Figure 6C). Moreover, the proportion of abnormal mitochondrial cristae and average circularity was not significantly different from those of the control group (Figure 6D,E). There was also no significant difference in the mRNA levels of fatty acid composition- and mitochondrial function-related genes between these two groups (Figure 6F). These results indicate ELOVL6 is an important target gene of miR-22 in the regulation of fatty acid composition and mitochondrial morphology in C2C12 cells.

## 4. Discussion

MiR-22 is highly conserved across diverse species and ubiquitously expressed in various tissues, and it is particularly highly expressed in skeletal muscle and adipose tissue [23]. This study revealed that miR-22 plays a pivotal role in the fatty acid composition and mitochondrial morphology in muscle cells. Previous studies have shown that miR-22 represses fatty acid synthesis and elongation of endogenous palmitate in tumor cells [24]. In the present study, miR-22 was discovered to reduce the elongation of endogenous palmitic acid (C16:0) to oleic acid (C18:1) and linoleic acid (C18:2) in C2C12 cells. PPARα signaling pathway genes were regulators of lipid metabolism, and they were negatively associated with saturated fatty acid (SFA) deposition in muscle [25,26]. PPARα was downregulated and upregulated when miR-22 was overexpressed and inhibited, respectively, in C2C12 cells, indicating that miR-22 might also play a role in the regulation of muscle fatty acid composition. Linoleic acid (C18:2) plays a stimulatory role in glucose uptake in differentiated C2C12 skeletal muscle cells [27]. The inhibition of fatty acid elongation by miR-22 may affect skeletal muscle glucose metabolism. Oleic acid (C18:1) and linoleic acid (C18:2) in pork are thought to improve meat flavor [28,29]. Moreover, high content of saturated fatty acids can significantly increase the redness of pork [30]. We suspect that its effects on fatty acid composition might be one way in which miR-22 affects meat quality. 

Mitochondria is the main oxygen consumer in muscles after slaughter. Mitochondrial quantity, shape, and function are closely related to meat quality [31]. Mitochondrial membrane potential reflects the integrity of mitochondria and is a sensitive indicator of mitochondrial function. Decreases in mitochondrial membrane potential lead to a decline in mitochondrial energy conversion and oxidative phosphorylation, among other functions [32]. The mitochondrial membrane potential in C2C12 cells was inhibited by miR-22 overexpression and increased by miR-22 inhibition in this study. Moreover, injecting miR-22 inhibitors into the rat myocardium also increased mitochondrial membrane potential and ATP production, thereby protecting myocardial mitochondria from damage [33]. In addition, the results showed that miR-22 significantly inhibited the expression of Ppp3ca and TfR1, two important genes related to Ca^2+^ signal transmission. Downregulation of these genes could expedite the accumulation of Ca^2+^ in mitochondria, thereby interfering with mitochondrial membrane potential [34,35,36]. Electron microscopy revealed that the expression of miR-22 increased the proportion of abnormal mitochondrial cristae and promoted mitochondrial division in C2C12 cells. Abnormal cristae morphology negatively affects mitochondrial respiration and oxidative phosphorylation [37]. Mitochondrial function is closely related to their continuous fusion and fission, and fusion accelerates mitochondrial energy conversion [38]. Therefore, we suspect that miR-22 may play an important role in muscle cell mitochondria energy metabolism.

According to our findings, miR-22 significantly decreased the expression of RISP and NRF1, two genes related to mitochondrial oxidative metabolism. RISP is a functional component of mitochondrial complex III, which is important in electron transmission and mitochondrial oxidation [39]. NRF1 is mainly involved in mitochondrial biosynthesis and regulates the expression of certain subunits of the mitochondrial respiratory chain [40,41]. Previous studies have reported that mitochondrial oxidative metabolism is associated with the meat color and intramuscular fat content of pork [42]. Therefore, our results suggest that miR-22 may play an important role in meat quality by regulating mitochondrial function.

Through bioinformatics analysis and dual-luciferase assay, ELOVL6 was predicted and confirmed as a direct target of miR-22. Meanwhile, miR-22 significantly inhibited the expression of ELOVL6 in C2C12 muscle cells. Additionally, knockdown or overexpression of ELOVL6 could mimic the effect of miR-22 on fatty acid composition and mitochondrial function. In a previous study on the function of miR-22 in muscle, the expression of β-tubulin was stable and was used as a housekeeping protein [43]. Thus, β-tubulin was preferred in the present study. Previous works have also reported that ELOVL6 is a target gene of miR-22, and is also involved in the fatty acid synthesis and the elongation of liver cells and tumor cells [14,24]. 

Our in vitro experiments suggested that ELOVL6 could catalyze the extension of C16–C18 fatty acids in muscle cells. Coincidentally, a similar effect on fatty acid elongation was observed in the lung tissue of ELOVL6 knockout mice [44]. The expression of PPARα was decreased when that of ELOVL6 was altered in C2C12 cells. Similarly, PPARα decreased considerably in the liver of ELOVL6^−/−^ mice [45]. Therefore, PPARα may be an important gene through which miR-22 targets ELOVL6 to regulate fatty acid composition. However, PPARα is also a direct target of miR-22 [46]. Whether miR-22 directly or indirectly affects PPARα expression in muscle cells requires further investigation. 

The membrane potential of C2C12 cell mitochondria declined after knocking down ELOVL6, and mitochondria with vacuoles and abnormal cristae were observed and mitochondrial division increased. A previous study confirmed that mitochondrial respiratory function is closely related to mitochondrial membrane potential [47]. In Drosophila with mutations in the ELOVL6 gene, mitochondrial respiratory function was shown to be impaired [13]. At the same time, knocking out the ELOVL6 gene in Drosophila reduced the stearic acid content in mitochondria and inhibited the expression of the TfR1 gene, thereby repressing mitochondrial fusion and function [13]. Our results showed that ELOVL6 could promote the expression of mitochondrial function-related genes (RISP, Ppp3ca, NRFl, and TfR1) in C2C12 cells. Thus, it is likely ELOVL6 also affects mitochondrial function in muscle.

Furthermore, it was found that the overexpression of ELOVL6 could reverse a series of functional changes caused by miR-22. These findings not only demonstrated that ELOVL6 is a target gene of miR-22 in C2C12 cells, but also that it is an important gene downstream of miR-22 that affects muscle fatty acid composition and mitochondrial morphology. The regulatory mechanism of muscle fatty acid composition and mitochondrial function is complicated, and we have only conducted a preliminary study on the role of miR-22 in vitro. In cancer cells, miR-22 targets the myelocytomatosis oncogene (MYC) gene to regulate fatty acid metabolism, and MYC is highly expressed during muscle development [24,48]. miR-22 can also inhibit the LIG3 gene and affect cancer cell mitochondrial structure and function [49]. LIG3 plays an important role in maintaining the integrity and function of mitochondrial DNA [50]. In the future, additional studies were needed to further demonstrate the regulatory effect of miR-22 on skeletal muscle fatty acid composition and mitochondrial function in farm animals.

## 5. Conclusions

The findings demonstrated that miR-22 increased the relative content of palmitic acid (C16:0) in C2C12 muscle cells and reduced oleic acid (C18:1) and linoleic acid (C18:2) by inhibiting the expression of ELOVL6. It also reduced the mitochondrial membrane potential of muscle cells, while it promoted cell mitochondrial division and the appearance of abnormal cristae by targeting ELOVL6. Additionally, miR-22 targeted ELOVL6 to inhibit the expression of important genes related to fatty acid composition and mitochondrial function. In conclusion, this study provides evidence of the mechanism through which miR-22 represses fatty acid elongation and affects the mitochondrial morphology of muscle cells by targeting ELOVL6.

## Figures and Tables

**Table 1:** All primers used in this study.

Gene Name	Sequence (5′-3′)	Accession Number
GAPDH	F: ATCACTGCCACCCAGAAGACT	NM_008084.2
	R: CATGCCAGTGAGCTTCCCGTT	
ELOVL6	F: GAAAAGCAGTTCAACGAGAACG	NM_130450.2
	R: AGATGCCGACCACCAAAGATA	
PPARa	F: AGAGCCCCATCTGTCCTCTC	NM_011144.6
	R: ACTGGTAGTCTGCAAAACCAAA	
RISP	F: CTTCTGTCCGTTTTTCCC	NM_025710.2
	R: GGTTTGCCTCTCCATTTA	
NRF1	F: GCCGTCGGAGCACTTACT	NM_001164226.1
	R: CTGTTCCAAGGTCACCACC	
Ppp3ca	F: TGTACACGGTGGTTTGTCTCCAG	NM_001293622.1
	R: GGCCCATAAGCAGGTGGTTC	
TfR1	F: GCAGCTATTGCACTAGTC	NM_011638.4
	R: TGACTGCACTATGGTCAC	
U6	F: GTGCTCGCTTCGGCAGCACATAT	NR_003027.2
	R: AAAATATGGAACGCTTCACGAA	
miR-22	F: CAGGAAGCTGCCAGTTGAA	NR_030711.1
	R: TCAACTGGTGTCGTGGAGTC	
RT-loop-miR-22	CTCAACTGGTGTCGTGGAGTCGGCAATTCAGTTGAGACAGTTC	

Note: GAPDH, glyceraldehyde-3-phosphate dehydrogenase; ELOVL6, elongation of long-chain fatty acids family member 6; PPARa, peroxisome proliferator activated receptor alpha; RISP, rieske iron-sulfur polypeptide 1; NRF1, nuclear respiratory factor 1; Ppp3ca, protein phosphatase 3 catalytic subunit alpha isoform; TfR1, transferrin receptor; U6, U6 small nuclear RNA.

## Data Availability

The data presented in this study are available on request from the corresponding author.

## Supplementary Materials

The following supporting information can be downloaded at: https://www.mdpi.com/article/10.3390/ani12172274/s1, Table S1: Sequence of RNA Oligonucleotides.
