# Supplementary material for: A Specific microRNA Targets an Elongase of Very Long Chain Fatty Acids to Regulate Fatty Acid Composition and Mitochondrial Morphology of Skeletal Muscle Cells
Source: Animals (Basel). 2022 Sep 2;12(17):2274. doi: 10.3390/ani12172274 (PMC9454801; doi:10.3390/ani12172274)
Supplement: Supplementary file 1 [file animals-12-02274-s001.zip › animals-1840690-supplementary.pdf]

Supplementary Material

Table S1 –Sequence of RNA Oligonucleotides

**Name**	**Forward (5’-3’)**	**Reverse (5’-3’)**
**RNA oligonucleotides**		
Negative control	UUCUCCGAACGUGUCACGUdTdT	ACGUGACACGUUCGGAGAAdTdT
miR-22-mimics	AAGCUGCCAGUUGAAGAACUGU	AGUUCUUCAACUGGCAGCUUUU
miR-22-inhibitor	CAGUUCUUCAACUGGCAGCUU	
Inhibitor NC	UCUACUCUUUCUAGGAGGUUGUGA	
